# Mechanical properties of the rabbit and human decellularized patches for well-tolerated/reinforced organ in cardiac tissue engineering

**DOI:** 10.34172/jcvtr.2023.32926

**Published:** 2023-12-30

**Authors:** Maryam Mousavi Khatat, Saeideh Same, Keyvan Moharamzadeh, Jafar Soleimani Rad, Ahmad Mehdipour, Leila Roshangar

**Affiliations:** ^1^Department of Tissue Engineering, Faculty of Advanced Medical Sciences, Tabriz University of Medical Sciences, Tabriz, Iran; ^2^Research Center of Pharmaceutical Nanotechnology, Tabriz University of Medical Sciences, Tabriz, Iran; ^3^Hamdan Bin Mohammed College of Dental Medicine (HBMCDM), Mohammed Bin Rashid University of Medicine and Health Sciences (MBRU), Dubai, United Arab Emirates; ^4^Department of Anatomy and Histology, Tabriz University of Medical Sciences, Tabriz, Iran

**Keywords:** Tensile strength, Mechanical strength, Tissue engineering, Decellular patch, Cardiac patches

## Abstract

**Introduction:**

Natural decellularized patches have been developed as the therapeutic platform for the treatment of different diseases, especially cardiovascular disorders. Decellularized scaffolds (as both cell-seeded and cell-free patches) are broadly studied in heart tissue redevelopment in vivo and in vitro. The designed regenerative bio-scaffold must have desirable physicochemical properties including mechanical stiffness for load-bearing, and appropriate anatomical characteristics to mimic the native biological environment properly and facilitate tissue reconstruction. In this context, the current study was designed to investigate rabbit decellularized derma’s similarity with human decellularized skin in terms of mechanical properties for cardiac tissue engineering application.

**Methods:**

Fifty two rabbit dermal specimens were provided and divided into two groups: the experimental (decellularized) group and the control (group). Similarly, twelve human skin specimens were divided into the experimental (decellularized) and control groups. Initially, the effect of decellularization on the mechanical performance of scaffolds was analyzed. Then, the mechanical strength of decellularized rabbit skin was compared to decellularized human derma by measuring the stress strain and Young’s modulus of the samples.

**Results:**

The results showed that rabbit decellularized skin has a similar elastic range to human decellularized skin, despite being more elastic (*P*>0.05). In addition, after decellularization, both rabbit and human skin showed a non-significant decrease in elasticity (*P*>0.05). It is worth noting that the elasticity reduction in rabbit samples after skin decellularization was lower than in human samples.

**Conclusion:**

According to the results of this study and the similarities of rabbit decellularized derm to human skin and its advantages over it, along with the biological complexity of native cardiac ECM, this scaffold can be used as an alternative matrix for tissue-engineered cardiac patches.

## Introduction

 Millions of people across the globe are still at risk of heart disease, which continues to be a major health concern. Cardiomyocytes (CMs) in the adult mammalian heart lack the ability to regenerate and therefore cannot recover from ischemic injuries such as myocardial infarction (MI). Suffered from irreversible cardiac muscle death, CMs are replaced by fibrotic scar tissues over time. The loss of contractile capacity can lead to heart dysfunction and eventual failure. Developing new methods for regenerating the myocardium remains a difficult challenge. Cardiac tissue engineering is a novel approach to address organ shortages for transplants and heart disease treatment. Extensive studies have shown that cardiac patches can potentially restore cardiac function in a clinical setting. Patches consist of two parts: Drugs and scaffolds.^1^

 Scaffolds provide physical and structural support for cell adhesion and tissue growth, requiring proper mechanical and hemodynamic performance and durability.^2^

 Scaffolds can be made from either natural or synthetic materials. Although natural-origin scaffolds have superior cell substrate and biomolecule release performance, they generally lack optimal elasticity. Currently, natural scaffolds are derived from sources such as collagen, fibrin, and decellularized extracellular matrices.^2^

 The fabrication of proper decellularized patches possessing the required mechanical and biological features has been addressed as an effective therapeutic approach for the targeted healing of damaged cardiac tissue. Recent studies demonstrated that the implantation of human- or animal-derived decellularized heart patches has stimulated the regeneration of cardiac tissue.^3^ Cardiomyocytes (CMs) maturation could be affected by many factors, such as mechanical forces, remodeling of ECM, and electrical stimulation.^4^ Previous studies emphasized that designed cardiac patches, especially small-scale patches, might not support sufficient mechanical or structural strength for sufficient cell promotion, which is necessary for successful clinical translation.^5^

 Decellularization of non-autograft tissues has become very important in recent years. Removal of immunogenic cellular factors, preserving natural proteins and their protected genes in bio-matrix, and promotion of the possibility of cell differentiation to target cells are significant advantages of natural decellularized scaffolds that increase the rate of successful scaffold transplantation by downregulating the immune system stimulation against transplanted tissue and subsequent rejection.^6,7^ Also decellularized scaffolds do not have the problems of other synthetic scaffolds, such as the lack of biological complexity of the native cardiac ECM or the high cost of preparation. These biological decellularized scaffolds could be supplied from various tissues supply such as the skin dermis, small intestine, liver and bladder.^8,9^ Moreover, the mechanical properties of xenografts must be similar to the targeted tissue. The acellular dermal matrix (ADM) can be considered a new membrane for reconstructing mucosal defects.^10^ In a study, the efficacy and safety of ADM in patients with diabetic foot ulcers were evaluated. The results revealed that the wound healed completely, and the mean healing time was significantly shorter compared to standard care while the side effects were not significantly.^11^ In addition, Chen et al. found that severe burns could be treated with a dermal decellularized matrix which led to the commercialization of product.^12^ Importantly, studies that were subjected to applying the decellularized template have even been approved in pre-clinical trials and have been marketed as OASIS (small intestine of pigs) products.^13,14^ Despite ADM remarkable advantageous features for regeneration application, its optimal employment requires more in vivo animal and clinical studies.^14^ Selecting proper animal models and clinical studies could answer the suitability of designed bio-scaffold for effective transplantation more accurately.

 As mentioned it is crucial to maintain the optimal stretch and mechanical tolerance of decellular scaffolds; There are studies that used the dermis as cardiac patches.^15^ However, due to its insufficient stretch, it has to be combined with synthetic scaffolds to form a composite patch. It is worth noting that rabbit skin is more elastic mechanically when compared to human or pig skin, and it appears that there is no existing study that has utilized decellularized rabbit skin as a cardiac patch material.

**Figure 1:**
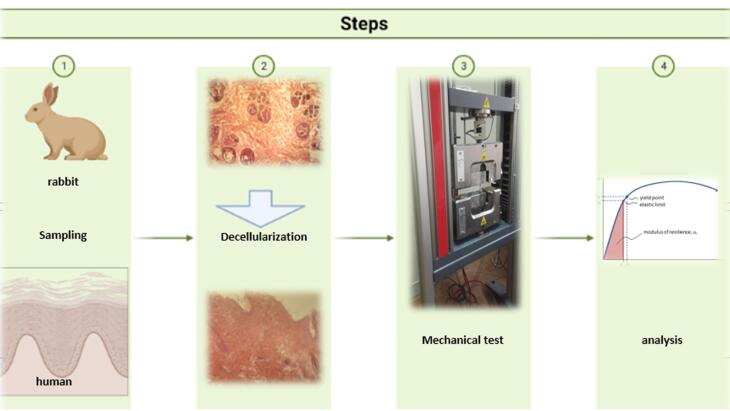
Schematic illustration of the project process briefly. After sampling and decellularization of the rabbit and human skin, the mechanical strengths of the prepared specimens were determined and analyzed

 Referring to the high value of global rabbit marketing and industrial usage, the application of rabbit skin seems to be a suitable candidate for preparing skin bio-scaffolds with properties resembling those of human skins for tissue engineering. The bio-based platform designed for application in regenerative medicine must have appropriate physicochemical, mechanical, and anatomical potential to simulate the natural biological environment and support tissue redevelopment.^16^ In this context, the present study was planned to determine whether rabbit skin could serve as an alternative substitute for human tissue studies in terms of mechanical properties for clinical regenerative therapies. Therefore, the comparative effects of stretching between decellular rabbit skin tissue and human decellular skin were investigated. The schematic steps of performing the current work for analyzing the mechanical properties of rabbit and human skin samples are disclosed in Figure 1.

## Materials and Methods

 For Sample preparation, Dutch Belted rabbits weighing about 2.2 to 2.5 Kg were used to sample the rabbit’s skin. Sampling was performed two hours after the euthanization of rabbits. In total, 52 rabbit dermal samples were extracted from the back and sides of three rabbits. Then, the biopsied samples were cut into smaller pieces (5 × 5 cm), and half of them were entered into the decellularization process, randomly as the experimental group.

 Human skin specimens were provided from the surplus skin in forensic medicine under the permission of the University Ethics Committee. The deceased candidates were aged 25 to 80 years, and regarding the limitation in size and number of the samples, only 12 human samples were prepared from the chest area. Similarly, some of them were run into the decellularization process, and the others were grouped as the controls.

 The decellularization procedure was carried out according to the Tissue Engineering Laboratory of Tabriz University of Medical Sciences protocol. Briefly, the epidermis was removed, and the prepared decellularized dermis was soaked in PBS solution for 24 hours. The descriptive histology analysis of the decellularized samples was carried out upon Hematoxylin and Eosin staining (H&E staining). The decellularized specimens were kept in a fixation solution for 48 hours, embedded in paraffin, and then dehydrated with a graded alcohol series (50%, 70%, 90%, 96%, 100%) followed by sectioning steps. Finally, the sectioned tissues were stained with H&E staining and visualized with light microscopy imaging to observe the remained cell nuclei.

 The stress-strain of decellularized sample tissues was determined using a Zwick tensile testing machine (Zwick / Roell, Z010) for four groups: decellularized rabbit dermis (26 samples), the control (26 samples: rabbit skin), the decellularized human dermis (six samples), and the control (six samples: human dermis). Each specimen was cut into 1 × 5 cm in length and fastened to two fixing clamps of the device. The test was carried out at an extension rate of 5 mm/min in the wet state. Values are mean ± SD (n = 3).

###  Statistical analysis

 Statistical analysis was performed to evaluate the difference between the mean tensions of the control group and decellular samples and to examine the difference between the mean tension of rabbit and human dermis using independent sample unpaired t-test. Analyzes were performed using GraphPad Prism software version 9. The 95% confidence interval was considered a statistical significance level.

## Results

**Figure 2:**
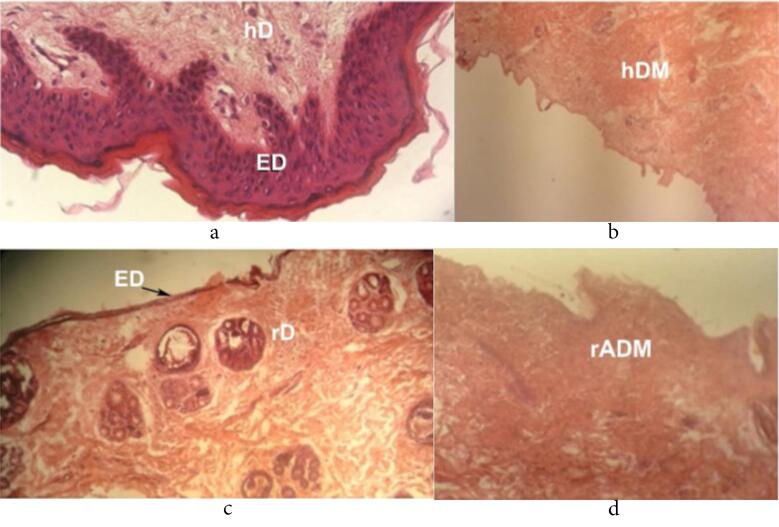
Optical micrograph of Hematoxylin&Eosin staining of human specimens (a × 400 & b × 100 ) and rabbit specimens (c & d: × 40). a) Human Dermis (hD) and Epidermis (ED) before decellularization that Cells are visible. b) Human Acellular Dermal Matrix (hADM) after decellularization that Cells have been removed. c) Rabbit Dermis (rD) and epidermis (ED) before decellularization that Cells are visible. d) Rabbit Acellular Dermal Matrix (rADM) after decellularization that Cells have been removed.

 Histological analysis based on decellularization protocol showed that the applied method could remove a significant percentage of skin cells in both human and rabbit samples. According to Figure 2, by maintaining the scaffold, the cells that are influential in transplant rejection were effectively removed.

**Figure 3:**
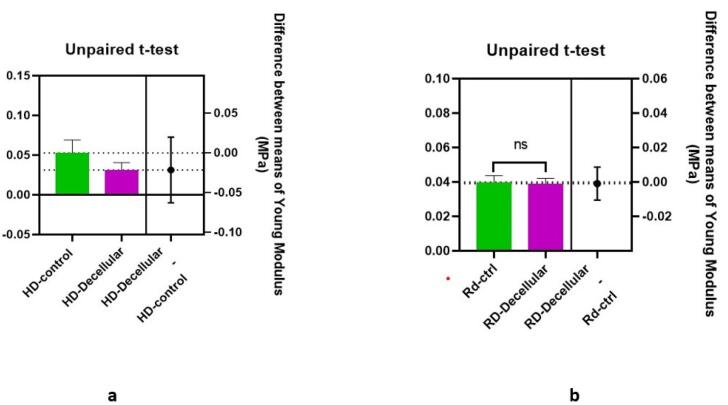
Unpaired sample t-test results of mean Young modulus in a) humans (HD) and b) rabbit (RD) skin groups (*P* > 0.05)

**Figure 4:**
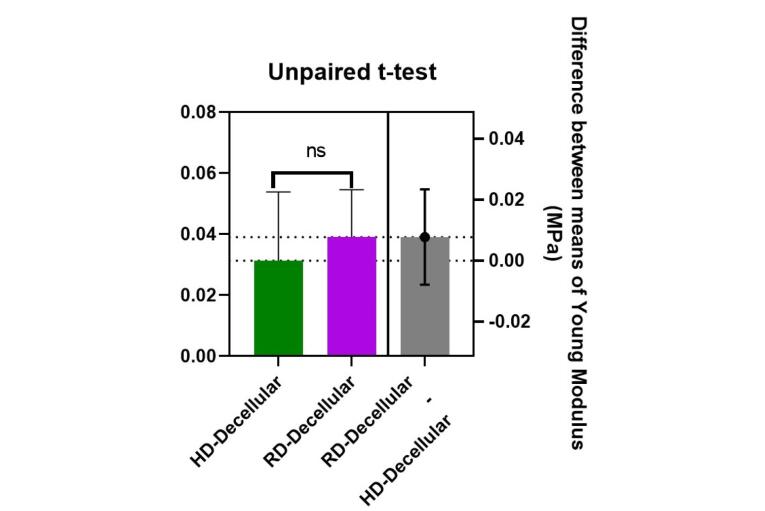
Independent sample unpaired t-test results of mean Young modulus between humans (HD) and rabbit (RD) skin groups (*P* > 0.05)

 Young modulus was calculated for both control and decellularized groups in rabbits and humans. The data normality assumption was confirmed via the Kolmogorov-Smirnov test (*P* > 0.05). The result disclosed in Table 1 showed Mean Young’s modulus values obtained for decellularized derm of humans and rabbits and their control groups. In addition, to evaluate the difference between the mean tensions of the control group and decellular samples and to examine the difference between the mean tension of rabbit and human dermis used independent sample unpaired t-test. The results showed that the mean elasticity in the decellular skin groups of rabbit and human were lower than in their control group, but this change was not significant (*P* = 0.847 for rabbit and *P* = 0.275 for human). These results showed that decellularization had no significant effect on the elasticity of the groups (Figure 3b).To evaluate the difference in elasticity (tension) in rabbit and human decellularized scaffolds, Young’s modulus was calculated and compared. The results (Figure 4) confirmed that there was not any significant (*P* = 0.373) difference between the decellular dermis of human and rabbit, in spite of the lower mean elasticity in the rabbit decellular skin than human decellular dermis.

**Table 1:** Mean Young's modulus values obtained for decellularized derm of humans and rabbits

**Sample**	**Rabbit**		**Human**	
	**Control**	**Decellular**	**Control**	**Decellular**
Young Modulus (MPa)	0.047 ± 0.023	0.038 ± 0.015	0.052 ± 0.039	0.031 ± 0.022

## Discussion

 The bio-based platform designed for application in regenerative medicine must have appropriate physicochemical and anatomical potential to simulate the natural biological environment and support tissue redevelopment.^16,17^ In this context, the current study was designed to investigate the similarity of rabbit decellularized derma with human decellularized skin in terms of mechanical strength for tissue engineering application. This study attempted to investigate the extent of rabbit skin elasticity as an alternative specimen in tissue engineering applications.

 The most important result of the present study was that the stretch mean between decellularized human and rabbit skin did not show significant differences. Although a significant difference was not obtained, the amount of elasticity in decellularized rabbit skin was found to be higher. In addition, the similarity of tensile properties of rabbit and human decellular skin scaffolds was also compared.

 The results revealed that applying decellularization process following the same method for both rabbit and human tissues reduced the amount of elasticity in both human and rabbit species. Although the difference was not significant, the elasticity decrease in rabbit decellularized skin was less than human skin.

 The previous research reported different and changeable results regarding rabbit skin Young’s modulus and human skin Young’s modulus. The different tissue and biological properties including a person’s genetics and age, and the nature of the skin and the test conditions could be some of the main reasons for this alternation. For example, Koene et al. reported that Young’s modulus of human skin ranged from 0.02 to 0.3 MPa.^18^ Park et al also, reported a range of 0.006 to 0.0289 MPa for the Young modulus.^19^ These data suggest that the results obtained from the current study are in the reported and usual range. However, Jansen & Rottier’s reported a different Young’s modulus range (from 1.07 to 2.157 MPa) in comparison to the result of the present study which resulted in 0.052 ± 0.039 Young’s modulus for human skin.^20^ The alteration between these obtained values may be related to the different experimental conditions and variable equipment that were applied during the studies. In addition, the research performed by Jansen & Rottier’s research was conducted in 1958 with non-standardized samples where the sterilization process may have a sensible effect on the mechanical strength of scaffolds.^21^

 Also, the duration of time elapsed after sampling was not considered and mentioned by these researchers. The time interval of sampling postmortem and sample separations from the primary tissue can affect the amount of skin elasticity.^22,23^ The current research work has attempted to perform as quickly as possible after sampling, and moisture of the samples was provided during the testing aiming to respond to the sensitivity of the applied tissue specimens to drought. A few studies have been considered or reported to fulfill these mentioned cases previously.

 In this study, although we introduced that rabbit-ADM has a similarity to human-ADM in terms of elasticity (young modulus), there are limitations worth noting. According to research reports, various factors can affect the amount of skin elasticity, including the location and position of the sampling which could reduce the possibility of generalization. of the results.^24,25^ In this study, to limit the effect of this factor, all human samples were collected from almost a single position.

 Also, there are not any underlying tissues such as subcutaneous fat in the rabbit skin, which is a relatively thick layer in the human dermis. The literature evidenced that subcutaneous fat could affect the rate of skin contraction.^20^ Therefore, subcutaneous fat was removed to evaluate only the dermis by providing similarity between both species. Future research s should focus on the investigation of the biocompatibility effects of this animal scaffold on a cellular basis in order to provide the all required parameters for replacing this prepared tissue template with tissue-engineered scaffolds.

## Conclusion

 The study revealed that rabbit decellularized skin has a similar elastic range to human decellularized skin, despite being more elastic. In addition, after decellularization, both rabbit and human skin showed a non-significant decrease in elasticity. It is worth noting that the elasticity reduction in rabbit samples after skin decellularization was lower than in human samples. Hence, due to its similarities to human skin and its advantages over it, along with its biological complexity of the native cardiac ECM, this scaffold can be implemented as an alternative matrix for tissue engineering investigations.

## Ethical Approval

 Necessary permissions to conduct research were obtained from the ethics committee of Tabriz University of Medical Sciences (IR.TBZMED.REC.1397.143). The research was conducted in accordance with the Declaration of Helsinki.

## Competing Interests

 Authors declare that they have no conflict of interest.

## Funding

 This study was funded by Tabriz medical University of Sciences (Grant number: IR.TBZMED.REC.1397.143).
